# Literary Notes

**Published:** 1903-02

**Authors:** 


### LITERARY NOTES.

THE TRIBUNE REVIEW.—*The Tribune Review* is quoted with respect by medical journals, and the accuracy of its editorial statements on scientific matters is attested by so high an authority as Lord Kelvin; while Prof. W. T. Spillman, the discoverer of Mendel's law, says that "the reason *The Tribune* is quoted so widely, and especially on scientific subjects, is that it is invariably so accurate."

*The Review* gathers and sifts every week the world's history, and serves it up with lucid comment. This publication, for the subscription price of $1 a year, places at the reader's service the trained abilities of the corps of correspondents (in this and foreign countries), editorial writers, literary, musical, art and dramatic critics, who coöperate in the making of a great metropolitan daily paper, which would cost you $10 a year if you had it delivered to you every morning.

*The Review* is an educational paper in the best sense. Not only are its general features such as to make it an admirable adjunct in the formation of character and opinion, but it contains special features of interest to parents and teachers. For example, a series of articles, entitled, "Careers for Young

Americans" is now running in its pages, including the following papers: Railroading, by George H. Daniels, general passenger agent of the New York Central Railroad; Journalism, by St. Clair McKelway, editor of *The Brooklyn Eagle*; The stage, by A. M. Palmer; Architecture, by Thomas Hastings, of Carrère & Hastings; Engineering, by a prominent engineer; Medicine, by Dr. D. B. St. John Roosa, president of the Post-Graduate Medical School and Hospital, New York City.

---

*La Revue Médicale du Canada* devotes practically its entire number, dated December 10, 1902, to a commemoration of the 25th Anniversary of Laval University. In this special number *La Revue* gives a history of the university with photographic reproductions of the university buildings and hospitals, together with biographical sketches of the faculty and pictures of each member. It is a creditable exhibition of journalistic enterprise upon which our contemporary deserves congratulations.

---

THE *Journal of Cutaneous Diseases including Syphilis* is the modified title of the former Journal of Cutaneous and Genitourinary Diseases. It is under the editorial direction of a group of dermatologists residing in New York, Boston, Chicago and Philadelphia. It will therefore in the future be a strictly dermatological journal. It is handsomely printed and this first new number contains much valuable material.

---

THE *California State Journal of Medicine* is the organ of the Medical Society of the State of California, and made its appearance in November, 1902. It is published by the society in the place of a yearly volume of transactions.

---

THE *Trained Nurse and Hospital Review* for January, 1903, contains the text of a bill proposed to be introduced in the legislature during the present session in relation to licensing nurses. It deserves great scrutiny by all interested.

---

THE *Dallas Medical Journal*, published at Dallas, Texas, is a new candidate for professional favor that appeared in November, 1902. It is edited by Dr. Alfred Roulet and its initial number presents an excellent appearance.

*Medicine*, edited by Dr. Harold N. Moyer, of Chicago, has taken on a new form and will be published hereafter as a double column quarto, enlarged, and with a new dress. We confess to a predilection for the former shape of this excellent magazine, but we congratulate the distinguished editor upon the splendid success of *Medicine*.

---

THE *Bulletin*, a quarterly medical review, which has been published since 1892 by the American Medical Temperance Association, has been consolidated with the *Journal of Inebriety*. The later journal, which first appeared in 1876, under the editorial care of Dr. T. D. Crothers, of Hartford, Ct., was the first and is still the only medical periodical in the world devoted exclusively to the scientific study of the neuroses and psychoses of spirit and drug diseases.

---

THE *Medical Library Journal*, a quarterly devoted to medical libraries, history, bibliography and biography, made its initial appearance during January. It is published in Brooklyn, and is issued under the joint editorship of John S. Brownne and Albert T. Huntington. It contains a large amount of very interesting reading matter, is well illustrated, and makes a handsome appearance.

---

THE *Index Medicus* is a monthly classified record of the current medical literature of the world. Its publication has been revived and the second series is published by the Carnegie Institution, Washington, D. C. The editors are Drs. Robert Fletcher and Fielding H. Garrison. It publishes no advertisements and does not exchange with other journals. The subscription price (to be paid in advance) is $5.00 a year in the United States and Canada; in foreign countries 25 shillings or marks, 30 francs or lire. All communications relating to subscriptions must be addressed: Carnegie Institution, Washington, D.C. Letters relating to the editorial department should be addressed: Editors *Index Medicus*, Washington, D.C.

---

THE *Archives of Pediatrics* for January, 1903, has been issued. It is the first of the twentieth yearly volume of a journal which is the oldest in the English language devoted exclusively to the diseases of infants and children. During the coming months its high reputation will be fully maintained, and its readers may count upon receiving contributions from the leading pediatrists of the world.

---

Messrs. W. B. Saunders & Company announce to the profession that they have established a branch of their business in New York. They have secured a suite of rooms in the Fuller Building, located at Fifth Avenue, Broadway and 23d Street. Dr. Reed B. Granger, for many years managing editor of the *New York Medical Journal*, together with a representative who is thoroughly familiar with the methods of the Philadelphia house, will be connected with this new branch; and Mr. W. B. Saunders personally will divide his time between New York and Philadelphia.

Physicians visiting New York are cordially invited to make these conveniently appointed offices their headquarters, where they can receive and answer their correspondence, obtain an interesting panoramic view of the city from a most favorable point, and where they will always be courteously welcomed.

---

The Books of a Year.—The *Medical Book News*, in the four numbers thus far issued, has provided an interesting illustration of the really very small figure for which all the new books on medicine and allied sciences published in 1902 might be purchased, were anyone desirous of so doing.

In the July (initial) number, 79 American and English titles appeared under the heading "Recent Publications." These titles represent 31,232 reading pages and 5,473 illustrations. The net price for this wealth of medical literature is only $287.15.

The September number listed 84 titles, these standing for 33,209 pages of printed text and 9,731 illustrations. The net purchase price for these 84 works is $239.41.

An increase in titles is noted in the November number, which announces 105 new publications. A total of 41,229 reading pages, carrying 7,202 illustrations, is here offered for $305.20.

The present issue, too, runs rather heavy in point of number of recent publications listed, 95 being found. These listed volumes contribute 41,183 pages of reading matter and 6,436 illustrations, at a net purchase price of $313.95.

A grand totaling, therefore, shows that the 363 new works published within the last twelve months, representing 146,853 pages of reading with 28,842 illustrations, could be purchased for the comparatively small sum of $1,145.71, this being an average of but $3.15 per volume.

